# Sutureless Aortic Valve Replacement with the Perceval Plus^®^ Versus TAVI: A Propensity-Matched Analysis of Postoperative Outcomes, Hematological Dynamics, and Inflammatory Response

**DOI:** 10.3390/life16101606

**Published:** 2026-09-25

**Authors:** Umit Kahraman, Irem Demiray, Serkan Ertugay, Emrah Oguz, Oguz Yavuzgil, Hakan Posacioglu, Anıl Ziya Apaydin, Ismet Tanzer Calkavur, Mustafa Ozbaran, Tahir Yagdi

**Affiliations:** 1 Department of Cardiovascular Surgery, Faculty of Medicine, Ege University, 35100 İzmir, Turkey; 2 Department of Cardiology, Faculty of Medicine, Ege University, 35100 İzmir, Turkey

**Keywords:** transcatheter aortic valve replacement, sutureless bioprosthesis, thrombocytopenia

## Abstract

Perceval Plus and TAVI are increasingly used for severe aortic stenosis, both relying on a self-expanding nitinol stent design, yet evidence specific to Perceval Plus remains limited regarding postoperative clinical outcomes and hematological and inflammatory dynamics. We compared postoperative clinical outcomes and longitudinal platelet/CRP dynamics between Perceval Plus and TAVI in a retrospective single-center cohort, in which 26 propensity score-matched pairs were obtained (1:1 matching) from patients undergoing Su-AVR (Perceval Plus, *n* = 53) or TAVI (self-expanding Medtronic devices, *n* = 101). Mortality, echocardiographic outcomes, and rates of postoperative atrial fibrillation and conduction disturbances showed no statistically significant differences between groups, while TAVI was associated with shorter ICU and hospital stay and Su-AVR required higher FFP transfusion (*p* = 0.016). Platelet count declined in both groups, with a deeper nadir in Su-AVR (postoperative day 3: 66% decline) versus TAVI (day 4: 27% decline), with a significant group × time interaction (*p* = 0.013); CRP rose in both groups but more rapidly and to a greater magnitude in Su-AVR (time and group × time interaction, both *p* < 0.001). Su-AVR and TAVI showed no statistically significant differences in the clinical safety outcomes assessed but differed in postoperative hematological and inflammatory magnitude, likely reflecting multifactorial procedure- and device-related mechanisms, warranting confirmation in larger, prospective, multicenter studies.

## 1. Introduction

Severe aortic stenosis (AS) is the most prevalent valvular heart disease in the elderly population of Europe and North America, with a prevalence reaching 3.4% in individuals over 75 years of age; the Turkish Registry of Heart Valve Disease found aortic stenosis in 6% of patients with native valvular heart disease presenting to cardiology clinics [1,2]. Untreated symptomatic severe AS carries a mean survival of five years for angina, three for syncope, and two for congestive heart failure [3]. Over the past two decades, transcatheter technologies have expanded treatment indications, while sutureless valve technology has been developed to shorten cardiopulmonary bypass (CPB) duration and simplify implantation [4].

Conventional SAVR remains the gold standard for severe AS, supported by decades of evidence on durable hemodynamic correction and survival [5], but CPB, cardioplegic arrest, and full sternotomy have limited its use in high-risk, elderly, or comorbid patients. Sutureless bioprostheses, most notably the Perceval^®^ S (Corcym S.r.l., Saluggia, Italy), reduce this morbidity by eliminating individual suture placement, shortening operative time while preserving direct-visualization removal of the native valve [6], with favorable hemodynamic performance in multicenter registries, particularly in small annulus, porcelain aorta, or concomitant procedures [7,8,9]. The Perceval Plus^®^ (Corcym S.r.l., Saluggia, Italy), introduced in 2019, preserves this architecture while adding the FREE anticalcification treatment [10].

TAVI emerged in parallel as a less invasive alternative; in landmark trials across risk strata, it demonstrated non-inferiority or superiority to SAVR for mortality and disabling stroke [11,12,13]. It has since expanded into intermediate- and low-risk patients, as reflected in current guidelines [14].

Although procedurally distinct, Su-AVR with the Perceval Plus^®^ and TAVI share a fundamental commonality: both rely on a self-expanding nitinol framework for anchoring without conventional sutures [15,16]. Postoperative thrombocytopenia after sutureless valve implantation was first described by Albacker et al., with a significant, protracted platelet decline versus conventional sutured replacement [17], while self-expanding transcatheter prostheses have similarly been associated with thrombocytopenia in up to 95.8% of TAVI patients [18]. Whether this shared design independently contributes to platelet decline beyond bypass-related effects remains unclear; TAVI, performed without CPB, allows this distinction to be examined.

Beyond hematological considerations, the systemic inflammatory response triggered by CPB and the accompanying surgical trauma is an important source of postoperative morbidity [19]. TAVI, by avoiding it entirely, is associated with an attenuated inflammatory response, with transfemoral access showing the least pronounced elevation [20]. However, serial comparative data on both platelet and CRP trajectories between Su-AVR with the Perceval Plus^®^ and TAVI remain scarce.

In this context, the present study compares postoperative clinical outcomes, longitudinal platelet trajectories, and serial CRP levels between patients undergoing Su-AVR with the Perceval Plus^®^ bioprosthesis and those undergoing TAVI, using propensity score matching to minimize baseline confounding in a single-center cohort.

## 2. Materials and Methods

### 2.1. Study Design and Ethics Approval

This single-center, retrospective observational cohort study was conducted at Ege University Faculty of Medicine, İzmir, Turkey. The protocol was approved by the Institutional Ethics Committee of Ege University Faculty of Medicine (approval no. 26-3T/75; approval date: 5 March 2026) and conducted in accordance with the Declaration of Helsinki. Given the retrospective design and use of anonymized data, individual informed consent was waived.

### 2.2. Study Population

A total of 154 consecutive patients undergoing aortic valve replacement for severe aortic stenosis at our institution were retrospectively reviewed. Patients were divided into two groups: the sutureless aortic valve replacement (Su-AVR) group, comprising those who underwent surgical replacement with the Perceval Plus^®^ bioprosthesis (Corcym S.r.l., Saluggia, Italy) between September 2019 and December 2025 (*n* = 53); and the transcatheter aortic valve implantation (TAVI) group, comprising those treated at our Cardiology Angiography Laboratory jointly by Cardiovascular Surgery and Cardiology between October 2021 and December 2025 (*n* = 101). Exclusion criteria were predominant aortic regurgitation as the primary indication, emergency procedures, incomplete records, and active infective endocarditis.

### 2.3. Surgical and Transcatheter Procedures

Su-AVR patients underwent implantation of the Perceval Plus^®^ self-expanding sutureless bioprosthesis under cardiopulmonary bypass (CPB), via full median sternotomy or minimally invasive partial upper sternotomy. Prosthesis size (Small–Extra Large) was determined intraoperatively using dedicated sizing templates. One patient underwent concomitant coronary artery bypass grafting; no other concomitant procedures were performed. TAVI patients were treated transfemorally under joint Cardiovascular Surgery–Cardiology management; device selection (Medtronic CoreValve, Evolut R, or Evolut Pro; Medtronic, Minneapolis, MN, USA) and sizing were guided by preoperative CT angiography and Heart Team consensus.

### 2.4. Data Collection

Baseline demographic, laboratory, echocardiographic, and procedural data were retrieved retrospectively from institutional electronic records. Preoperative variables included age, sex, body surface area, diabetes, COPD, chronic renal failure, dialysis dependency, prior cardiac surgery, LVEF, atrial fibrillation, conduction abnormalities (LBBB, RBBB, pacemaker rhythm), pulmonary hypertension (systolic pulmonary artery pressure > 45 mmHg), EuroSCORE II, thrombocytopenia and its etiology, and hepatic comorbidities (cirrhosis, hypersplenism). Preoperative laboratory assessments included hemoglobin, hematocrit, leukocyte count, platelet count, albumin, AST, ALT, blood urea nitrogen, creatinine, INR, and CRP.

Postoperative outcomes included ICU and total hospital stay, 30-day and in-hospital mortality, new-onset atrial fibrillation, conduction disturbances requiring pacemaker, hemorrhagic and ischemic stroke, re-exploration for bleeding, low cardiac output syndrome (LCOS), respiratory insufficiency, gastrointestinal bleeding, and renal replacement therapy. Transfusion requirements (RBC, FFP, platelets), postoperative aortic gradients, paravalvular leakage, and anticoagulation/antiplatelet regimens were also recorded. Serial platelet counts and CRP levels were measured preoperatively and on postoperative days 1–7 and 14. In accordance with institutional practice, blood samples were drawn at a standardized time each morning (approximately 06:00) as part of routine postoperative laboratory monitoring, minimizing variability related to sampling time.

### 2.5. Statistical Analysis

Statistical analyses were performed using IBM SPSS Statistics for macOS, version 30.0 (IBM Corp., Armonk, NY, USA). The distribution of continuous variables was assessed using the Kolmogorov–Smirnov test, histograms, and skewness and kurtosis values. Continuous variables were presented as mean ± standard deviation or median (interquartile range, IQR), according to their distribution, and categorical variables as number and percentage [*n* (%)].

In the unmatched cohort, categorical variables were compared using the chi-square or Fisher’s exact test, and continuous variables using Student’s *t*-test or the Mann–Whitney *U* test, as appropriate. Covariate balance was assessed using standardized mean differences (SMDs). An absolute SMD ≥ 0.20 was considered indicative of potentially meaningful imbalance.

Propensity scores were estimated using a logistic regression model including age, sex, left ventricular ejection fraction (LVEF), EuroSCORE II, chronic renal failure, and chronic obstructive pulmonary disease (COPD). Patients were matched 1:1 using nearest-neighbor matching without replacement, yielding 26 matched pairs (*n* = 52). Post-matching balance was re-evaluated using SMD.

Within the matched cohort, categorical variables were compared using the exact McNemar test and continuous variables using the Wilcoxon signed-rank test. As an additional sensitivity analysis, an expanded propensity score model was constructed by adding baseline platelet count, leukocyte count, and CRP to the original propensity score covariates. Patients were matched 1:1 using nearest-neighbor matching without replacement and a caliper of 0.2 standard deviations of the logit of the propensity score, yielding 35 matched pairs. Covariate balance was assessed using SMDs. This expanded model was used as a sensitivity analysis to assess the robustness of the primary findings to residual imbalance in baseline hematological and inflammatory markers. In addition, baseline characteristics of matched and unmatched TAVI patients were compared using SMDs to assess potential selection associated with the matching procedure. Variables without observed variability, or for which comparison was not applicable, are reported descriptively (NA).

Postoperative changes in platelet and C-reactive protein (CRP) levels were analyzed using linear mixed-effects models. Group, time, and the group-by-time interaction were included as fixed effects, with time treated as a categorical variable. A subject-specific random intercept with a variance component covariance structure was included to account for within-subject correlation across repeated measurements. No additional residual covariance structure for repeated measurements was specified. The platelet model was adjusted for baseline platelet count. CRP values were logarithmically transformed, and the model was adjusted for baseline log-CRP. As a sensitivity analysis, inverse probability of treatment weighting (IPTW) was performed in the full cohort. Propensity scores were estimated using the same covariates as in the PSM analysis. Stabilized weights were truncated at the 1st and 99th percentiles, and covariate balance after weighting was assessed using SMDs. Longitudinal analyses were performed using weighted generalized estimating equations (GEE) with robust standard errors. In the GEE models, time was treated as a categorical variable; the platelet analysis was adjusted for baseline platelet count, and the log-transformed CRP analysis was adjusted for baseline log-CRP. All tests were two-sided, and *p* < 0.05 was considered statistically significant.

## 3. Results

### 3.1. Baseline Characteristics Before and After Propensity Score Matching

In the unmatched cohort, groups differed significantly in age, COPD, EuroSCORE II, LVEF, pulmonary hypertension, atrial fibrillation, hemoglobin, leukocyte count, albumin, and INR (all *p* < 0.05); complete baseline characteristics are presented in Table 1.

After 1:1 propensity score matching, 26 matched pairs were obtained. Balance was achieved (SMD < 0.2) for most variables, including age, LVEF, RBBB, paced rhythm, hypersplenism, and liver cirrhosis. However, SMD remained ≥0.2 for sex, diabetes mellitus, COPD, chronic renal failure, dialysis dependency, prior cardiac operation, EuroSCORE II, maximum and mean aortic valve gradient, pulmonary hypertension, atrial fibrillation, and preoperative thrombocytopenia; residual imbalance was also observed for hemoglobin, leukocyte count, hematocrit, platelet count, albumin, and CRP (SMD range, 0.34–0.69). All SMD values are presented in full in Table 2. As a sensitivity analysis, an expanded propensity score model incorporating baseline platelet count, leukocyte count, and CRP yielded 35 matched pairs with substantially improved balance on these variables (SMD 0.059, 0.074, and 0.081, respectively); the group × time interaction remained significant in this expanded cohort for both platelet count (*p* = 0.009) and CRP (*p* < 0.001), consistent with the primary matched-cohort analysis (Supplementary Table S1). Given that only 26 of 101 TAVI patients were retained after matching, baseline characteristics of the 26 matched and 75 unmatched TAVI patients were additionally compared using SMDs; the largest differences were observed for age, LVEF, EuroSCORE II, and COPD, indicating that the matched TAVI patients represented a selected subset of the original TAVI cohort (Supplementary Table S2).

### 3.2. Postoperative Clinical Outcomes

No significant between-group differences were observed in postoperative conduction disturbances, in-hospital or 30-day mortality, bleeding-related events, low cardiac output syndrome, respiratory insufficiency, gastrointestinal bleeding, renal replacement therapy, stroke, or paravalvular leak (all *p* ≥ 0.130 or not applicable); new-onset postoperative atrial fibrillation occurred more frequently in the Su-AVR group than in the TAVI group (19.2% vs. 0%), though this difference did not reach statistical significance (*p* = 0.063); complete data are presented in Table 3. ICU length of stay showed identical medians between groups (2 [2–2] vs. 2 [2–4] days) but was longer on average in the Su-AVR group (mean ± SD: 2.19 ± 1.06 vs. 4.62 ± 8.41 days, *p* = 0.041); total hospital length of stay was shorter in the TAVI group (6 [5–8] vs. 10 [8–12] days, *p* = 0.016). FFP transfusion requirement was higher in the Su-AVR group (*p* = 0.016); no significant difference was observed for RBC or platelet transfusion. 

### 3.3. Longitudinal Changes in Platelet Count and C-Reactive Protein

The number of available platelet and CRP measurements at each postoperative time point is provided in Supplementary Table S3; measurement availability was highest at baseline and decreased at later time points, consistent with variability in clinical follow-up sampling.

Platelet count declined in both groups postoperatively, with a more pronounced and earlier nadir in the Su-AVR group (postoperative day 3: 82.7 ± 29.2 × 10^9^/L, a 66% decline from baseline) compared with a later, less pronounced nadir in the TAVI group (postoperative day 4: 147.5 ± 49.4 × 10^9^/L, a 27% decline from baseline). Among patients with available POD14 measurements, platelet values were closer to baseline; however, measurement availability was limited at this time point. In the linear mixed-effects model adjusted for baseline platelet count, both the time effect (*p* < 0.001) and the group × time interaction (*p* = 0.013) were significant, confirming a divergent postoperative platelet trajectory between groups. Time-point-specific between-group differences are presented in Supplementary Table S4, and complete serial values are presented in Table 4 and Figure 1A.

CRP rose markedly in both groups, peaking earlier in the Su-AVR group (postoperative day 2: 156.5 [134.2–210.2] mg/L) and later, at a lower magnitude, in the TAVI group (day 3: 52.0 [14.0–70.5] mg/L). Among patients with available POD14 measurements, CRP values did not differ significantly between groups; however, measurement availability was limited at this time point. In the linear mixed-effects model adjusted for baseline log-CRP, both the time effect (*p* < 0.001) and the group × time interaction (*p* < 0.001) were significant, indicating a markedly greater and more rapid postoperative CRP rise in the Su-AVR group. As a sensitivity analysis, inverse probability of treatment weighting (IPTW) was performed using the full cohort (*N* = 154); the group × time interaction remained significant for both platelet count (*p* = 0.024) and CRP (*p* < 0.001), consistent with the primary matched-cohort analysis (Supplementary Table S5). Complete serial values are presented in Table 4 and Figure 1B. Baseline-adjusted, time-specific between-group differences are provided in Supplementary Table S4. Procedural details, valve sizes, and postoperative anticoagulation/antiplatelet regimens are presented in Table 5.

## 4. Discussion

Before matching, groups differed substantially, reflecting the real-world practice of reserving TAVI for older, higher-risk patients: TAVI patients were older (SMD = 0.98), with higher EuroSCORE II (SMD = 0.68), lower LVEF (SMD = 0.59), and higher rates of pulmonary hypertension (SMD = 0.47), atrial fibrillation (SMD = 0.42), and COPD (SMD = 0.48); hemoglobin and albumin were lower in TAVI (SMD = 0.60 and SMD = 0.53, respectively; all SMD ≥0.20)—underscoring the need for matching.

After matching, 26 pairs were obtained with substantially improved balance (age, LVEF SMD ≤ 0.08), though residual imbalance persisted for platelet count, leukocyte count, hemoglobin, CRP, pulmonary hypertension, and atrial fibrillation (SMD 0.43–0.69)—particularly relevant as platelet count and CRP are the study’s primary outcome measures, while leukocyte count and hemoglobin are closely related hematological parameters. This should be considered a potential source of residual confounding inherent to the observational design and limited sample size. To test whether this residual imbalance affected our results, we performed a sensitivity analysis using an expanded propensity score model that additionally incorporated baseline platelet count, leukocyte count, and CRP. This yielded 35 matched pairs with substantially improved balance on these variables (SMD 0.059–0.081), and the platelet and CRP group × time interactions remained significant in this expanded cohort (*p* = 0.009 and *p* < 0.001, respectively), indicating that our primary findings are not driven by this residual imbalance (Supplementary Table S1).

Thirty-day mortality was 3.8% in both groups; in-hospital mortality was 3.8% in TAVI and 7.7% in Su-AVR (both *p* = 1.000), the excess reflecting a single prolonged hospitalization rather than early procedural death—consistent with the moderate-risk profile and Stanitsa et al.’s comparable finding (4.1% vs. 3.0%) in a propensity-matched cohort [21].

New-onset postoperative AF occurred in 0 (0%) TAVI and 5 (19.2%) Su-AVR patients (*p* = 0.063); the three TAVI patients with postoperative AF had persistent pre-existing AF rather than new-onset events, whereas all Su-AVR cases were genuinely new-onset, consistent with higher new-onset AF after Su-AVR reported in a meta-analysis (RR 3.66; 95% CI 1.95–6.89) [22], likely from CPB’s inflammatory sequelae. Patient-level review confirmed that the postoperative LBBB cases (2 TAVI, 1 Su-AVR) occurred in the same patients who had preoperative LBBB, confirming persistence rather than new events, unlike literature-reported new-onset rates (23% Su-AVR, 16% TAVI, 4% conventional AVR) [23]. One Su-AVR patient developed a genuinely new complete AV block (3.8%) requiring a permanent pacemaker; although the literature suggests higher TAVI pacemaker rates (11.2% vs. 5.6%) [21], this single case may reflect patient-specific factors given the small sample. No RBBB was observed.

The shorter ICU and hospital stay with TAVI (*p* = 0.041, *p* = 0.016) are consistent with a meta-analysis reporting longer Su-AVR stays (mean 2.56 days; *p* < 0.01) [22], though that analysis found no significant ICU stay difference (*p* = 0.12)—our cohort’s significance appears driven by a wide dispersion in the Su-AVR group (mean ± SD: 4.62 ± 8.41 days) rather than a consistent shift, and given equal medians and a small-subgroup effect, should be interpreted cautiously rather than as a robust finding. Shorter TAVI hospitalization may relate to the absence of sternotomy and CPB, with implications for resource utilization in an elderly population with limited physiological reserve.

Comparisons between TF-TAVI and Su-AVR have generally assessed transfusion as overall bleeding rates rather than individual components. Santarpino et al., in a 79-pair analysis, found significantly higher RBC transfusion in the Su-AVR group (1.7 ± 2 vs. 0.3 ± 0.9 units, *p* < 0.001) [24]; in our study, RBC transfusion showed no statistically significant difference between groups (*p* = 0.134), possibly explained by our relatively short CPB duration and low re-exploration rate. FFP transfusion was significantly higher in the Su-AVR group (*p* = 0.016), while platelet transfusion did not differ (*p* = 0.317); this isolated FFP elevation may reflect CPB-related multifactorial coagulopathy (hemodilution, tissue factor/contact activation, residual heparin) [25]. Furthermore, the more frequent and higher-volume FFP transfusion in the Su-AVR group raises the possibility of a dilutional contribution to the observed platelet nadir; as FFP is typically administered intraoperatively or in the early postoperative period, this timing may coincide with the day 3 platelet decline. Given that dilutional thrombocytopenia after cardiac surgery may be proportional to the volume of blood products administered, FFP-related hemodilution may be considered a potential contributing factor. Low cardiac output syndrome occurred in one Su-AVR patient (3.8%) and no TAVI patients (*p* = 1.000); in a Perceval series, LCOS was similarly absent in the sutureless group (4.5% in the sutured cohort), with reduced cross-clamp time interpreted as protective [26]. This isolated case may reflect ischemia–reperfusion injury during cross-clamping, an exposure absent in TAVI; however, patient-specific factors cannot be excluded given the limited interpretive power of a single case.

No episodes of re-exploration for bleeding, respiratory insufficiency, or stroke (hemorrhagic or ischemic) were recorded in either group, reflecting favorable procedural safety of both approaches and consistent with comparable rates reported in propensity-matched series [27]. One TAVI patient experienced gastrointestinal bleeding (3.8%) and one Su-AVR patient required renal replacement therapy (3.8%; both *p* = 1.000)—consistent with known complication profiles (GI bleeding ~1% after TAVI [28]; renal dysfunction up to 2.7% after Perceval [6]).

Postoperative echocardiographic outcomes were excellent, with no statistically significant differences between groups: aortic valve gradients and paravalvular leak rates (11.5% vs. 3.8%, *p* = 0.625) did not differ, and all leaks were mild. Although higher TAVI paravalvular leak rates have been reported (23.4% vs. 2.5%, *p* < 0.001) [21], our null finding may reflect small sample size, exclusive self-expanding device use, and mild leak severity; comparable gradients may relate to predominant use of large-sized (29 mm) valves, consistent with a similar study finding no gradient difference in large-valve patients [21]. A similarly higher rate of paravalvular leak with TAVR relative to Perceval sutureless AVR has also been reported in a more recent propensity-matched analysis (11% vs. 0%, *p* < 0.001) [29], further supporting the reproducibility of this pattern despite our null finding.

Among Su-AVR patients, the surgical approach was full median sternotomy in 76.9% and minimally invasive partial upper sternotomy in 23.1%; the most frequently implanted valve size was Medium (50.0%). Median CPB time was 70.5 min and cross-clamp time was 48.5 min, comparable to values reported for the Perceval valve in a meta-analysis (CPB: 67.4 ± 20.2 min; ACC: 39.6 ± 14.2 min) and consistent with the operative efficiency of the sutureless technique versus conventional sutured SAVR (CPB: 93.2 ± 25.8 min; ACC: 66 ± 17.4 min) [6]. Most TAVI patients received the CoreValve (65.4%), with a median valve size of 29 mm, and all procedures were transfemoral. Postoperative anticoagulation differed markedly: most TAVI patients received no anticoagulation (88.5%), consistent with guideline recommendations [30]; 53.8% of Su-AVR patients received anticoagulation (predominantly warfarin, 23.1%, or rivaroxaban, 19.2%), reflecting guideline-recommended short-term therapy after surgical bioprosthesis implantation and, in a subset, additional indications such as postoperative AF. Antiplatelet therapy was administered in 92.3% of TAVI and 80% of Su-AVR patients; these differences may have influenced postoperative platelet dynamics.

In the matched cohort, preoperative platelet count was 203.3 ± 53.6 in the TAVI group and 240.8 ± 57.0 × 10^9^/L in the Su-AVR group. Both groups showed a marked postoperative decline, with the nadir on day 3 in the Su-AVR group (82.7 ± 29.2 × 10^9^/L, ~66% decline) and day 4 in the TAVI group (147.5 ± 49.4 × 10^9^/L, ~27% decline). Among patients with available POD14 measurements, platelet values were closer to baseline, and the between-group difference was not statistically significant (*p* = 0.740, Supplementary Table S4); however, the limited number of observations at this time point precludes firm conclusions regarding complete recovery.

Platelet values at nine time points were analyzed using a linear mixed-effects model adjusted for baseline platelet count; both the time effect (*p* < 0.001) and the group × time interaction (*p* = 0.013) were significant, consistent with a genuine divergence in postoperative platelet trajectories between groups rather than a chance finding. The trajectory in each group may have been shaped by a combination of procedure- and device-specific factors, discussed below.

The effect of CPB on platelet count is well documented; bypass leads to a decline exceeding what hemodilution from the priming fluid alone can explain, with mechanical trauma, circuit adhesion, and organ sequestration also contributing to the true drop in circulating platelet count [31]. This suggests the deeper decline in the Su-AVR group may relate to CPB, while the more limited decline in TAVI is consistent with the absence of CPB.

The Perceval Plus^®^ employs a tissue treatment approach distinct from the homocysteic acid (HCA)-based process used in the earlier-generation Perceval S. This FREE (Freedom from Residual free aldEhydEs) technology involves alcohol-based phospholipid removal, taurine-mediated aldehyde neutralization, and storage in an aldehyde-free solution, reducing phospholipid content by an average of 96% and eliminating the need for rinsing prior to implantation [32]. Although HCA has been proposed to contribute to platelet decline, this hypothesis was questioned when directly tested, with residual HCA found to be negligible [33].

Kim et al. reported that the Perceval valve was associated with significantly more severe postoperative thrombocytopenia than the Intuity (Edwards Lifesciences, Irvine, CA, USA) rapid-deployment valve (<50,000/μL in 56.5% vs. 6.7% of patients, *p* < 0.001) and a correspondingly higher platelet transfusion requirement (*p* = 0.008), attributing this to the valves’ distinct tissue treatment methods (ThermaFix vs. FREE), though they noted this association remains speculative [34]. The markedly deeper platelet decline observed with the Perceval valve relative to TAVI in our cohort parallels Kim et al.’s finding, suggesting that FREE technology may partly contribute to the thrombocytopenia pattern observed, though the association remains speculative.

CoreValve, Evolut R, and Evolut Pro leaflets are constructed from glutaraldehyde-fixated porcine pericardium and require saline rinsing prior to implantation. An earlier in vitro study demonstrated lower platelet adhesion to glutaraldehyde-fixed leaflets compared with native tissue [35]. A more recent study found relatively mild thrombocytopenia with the glutaraldehyde-based Evolut, whereas markedly more severe thrombocytopenia was seen with the formaldehyde-based Portico (Abbott, St. Paul, MN, USA) valve, largely mitigated by additional rinsing [36]. As our TAVI cohort consisted entirely of glutaraldehyde-based devices, the mild decline observed, though not fully explained by this alone, parallels these findings.

Both Perceval Plus^®^ and CoreValve/Evolut share a bare nitinol stent frame in direct contact with circulating blood. In vitro studies have shown that blood contact with bare nitinol reduces platelet count and elevates activation markers [37]; however, no significant increase in platelet activation was detected after CoreValve implantation [38]. Albacker suggested that the Perceval’s nitinol stent might contribute to platelet activation through endothelial damage, reporting a significant platelet drop with slow recovery in his own cohort [17]. As nitinol is shared by both devices and platelet decline was observed in both groups of our cohort, its contribution cannot be dismissed, though current evidence is insufficient to establish it as a sole or proven mechanism.

Beyond device-specific factors, the procedure itself may contribute to the decline observed in the TAVI group: vascular access through large-bore sheaths via endothelial damage/shear stress, iodinated contrast via effects on platelet function, and the procedural inflammatory response via enhanced platelet-leukocyte interactions [39]. The limited decline in our TAVI group may be related to these mechanisms.

In contrast to the pattern observed for platelets, CRP levels diverged markedly between groups. In the Su-AVR group, CRP rose to 43.0 (33.0–54.0) mg/L on day 1, peaked at 156.5 (134.2–210.2) mg/L on day 2, and remained elevated through days 3–4. In the TAVI group, the response remained more limited, increasing to 6.5 (2.6–12.2) mg/L on day 1 and peaking at 52.0 (14.0–70.5) mg/L on day 3. Among patients with available POD14 measurements, CRP levels were no longer significantly different between groups; however, the limited number of observations at this time point precludes firm conclusions regarding resolution of the inflammatory response.

The linear mixed-effects model, adjusted for baseline log-CRP, showed significant time (*p* < 0.001) and group × time interaction (*p* < 0.001) effects, indicating that the Su-AVR group experienced a substantially greater and more rapid postoperative CRP rise than the TAVI group, rather than a temporally parallel pattern at a different magnitude. The study compared CRP across nine time points up to postoperative day 14, offering a level of detail not provided by most prior studies; the longitudinal model’s adjustment for baseline log-CRP helps account for the residual preoperative imbalance observed after matching (SMD = 0.43, higher in the Su-AVR group). This suggests that the substantially greater postoperative CRP rise observed in the Su-AVR group is not solely attributable to this baseline imbalance.

These findings are consistent with the prior literature suggesting CPB contributes to the systemic inflammatory response syndrome (SIRS) in cardiac surgery via blood contact with the extracorporeal circuit, cross-clamp-related ischemia–reperfusion injury, and complement activation [40]. This complement activation reportedly has a second, prolonged phase—related to CRP and persisting through postoperative day 5 [19]—whose time course parallels the sustained CRP elevation in our Su-AVR cohort, partly related to the baseline CRP imbalance noted above. However, CRP may not relate linearly to CPB duration: a study comparing Perceval with a conventional sutured bioprosthesis found no CRP difference despite shorter CPB/cross-clamp times in the Perceval group, while IL-6, IL-8, and procalcitonin were higher in the conventional group [41], suggesting CRP is less sensitive to CPB duration than other acute-phase markers.

The postulated basis for the CRP elevation observed following TAVI relates to tissue-level processes triggered by the valve implantation procedure itself: endothelial injury during valve deployment and stent frame expansion may lead to release of proinflammatory microparticles, which may trigger leukocyte mobilization via pattern recognition receptors; a second plausible mechanism is ischemia–reperfusion injury from hypoperfusion during rapid pacing or major bleeding [42]. Consistent with these mechanisms, CRP following TAVI has been reported to peak at postoperative day 2–3, corresponding with the day 3 peak observed in the TAVI group in our study.

The magnitude of this inflammatory response may be influenced by clinical and procedural factors. According to Ruparelia et al., among clinical factors, only diabetes was associated with a significantly higher peak CRP; among procedural factors, pre-implantation balloon valvuloplasty and major vascular complications were significant, potentially reflecting release of calcium and tissue debris during native valve fracturing as an inflammatory trigger—consistent with the tissue injury mechanism discussed above. Valve type, post-dilatation, and residual aortic regurgitation had no effect [43]. Our TAVI cohort’s exclusive use of self-expanding valves is therefore not a confounding factor.

## 5. Limitations

The present study has several limitations that warrant acknowledgment. First, its retrospective, single-center design introduces a selection bias that propensity score matching can only partially mitigate. However, a sensitivity analysis using inverse probability of treatment weighting in the full cohort yielded consistent results (Supplementary Table S5), suggesting that the observed platelet and CRP trajectories were not solely attributable to the reduced sample size imposed by propensity score matching. Furthermore, comparison of matched and unmatched TAVI patients showed that the matched subset was younger and had a more favorable risk profile (lower EuroSCORE II, higher LVEF, less COPD) than the excluded TAVI patients (Supplementary Table S2), indicating that propensity score matching itself introduced an additional degree of selection; this should be considered when generalizing our findings to the broader TAVI population. Second, the small sample size of the matched cohort (26 pairs) limits statistical power and may affect the precision and generalizability of the observed effect estimates, warranting confirmation in larger cohorts. Third, residual confounding from variables not included in the propensity score model—including procedural complexity, operator experience, and anesthetic management—cannot be excluded. Fourth, several variables retained SMD ≥0.2 after matching, including prior cardiac surgery, dialysis dependency, baseline leukocyte count, hemoglobin, pulmonary hypertension, and atrial fibrillation, may have independently influenced the postoperative trajectories observed. Although baseline platelet count and CRP also showed residual imbalance, these were directly incorporated as covariates in the respective longitudinal outcome models (Table 4). The remaining variables were not added individually to the primary longitudinal outcome models in order to avoid further model expansion in the limited matched sample. Instead, their contribution to residual imbalance was addressed separately at the matching stage: a sensitivity analysis using an expanded propensity score model—which incorporated baseline platelet count, leukocyte count, and CRP as additional matching covariates—achieved substantially improved balance on these three variables, and the platelet and CRP group × time interactions remained significant in this expanded cohort (Supplementary Table S1). Fifth, generalizability is constrained by the specific prostheses employed—exclusively the Perceval Plus^®^ for Su-AVR and exclusively self-expanding Medtronic devices for TAVI—and results may not extend to balloon-expandable transcatheter valves or other sutureless bioprosthesis platforms. Sixth, the absence of data on heparin-induced thrombocytopenia antibodies, von Willebrand factor activity, platelet activation markers (e.g., P-selectin, platelet factor 4), perioperative heparin dosing, and other acute-phase markers such as IL-6, procalcitonin, or fibrinogen limits mechanistic interpretation of the hematological and inflammatory findings. Seventh, the marked differences in postoperative anticoagulant and antiplatelet regimens between groups—notably higher warfarin and rivaroxaban use in the Su-AVR group—may have independently influenced platelet dynamics. This variable was not separately modeled as a covariate because postoperative anticoagulant and antiplatelet use is determined after surgery and is inherently dependent on the type of valve implanted; as a post-treatment variable, it could not be validly incorporated into the propensity score or longitudinal outcome models, which rely on pre-treatment characteristics. Eighth, longitudinal platelet and CRP measurements were incomplete from postoperative day 3 onward, and the extent of missingness differed between groups (Supplementary Table S3). At POD14, platelet counts were available for 9 of 26 TAVI and 18 of 26 Su-AVR patients, and CRP values for 8 of 26 and 17 of 26, respectively. Although the linear mixed-effects models accommodated unbalanced repeated measurements, inference relies on the assumption that missing observations are missing at random conditional on the observed data, which cannot be verified in this retrospective dataset. Patients with laboratory measurements available at these later time points may have differed systematically from those without; therefore, findings from these time points should be interpreted cautiously. Ninth, platelet and CRP measurements were limited to postoperative day 14, precluding assessment of inflammatory resolution or delayed clinical events beyond this point. Finally, the study population spans a long enrollment period (September 2019–December 2025 for Su-AVR and October 2021–December 2025 for TAVI), during which changes in surgical technique, perioperative protocols, and TAVI device generations may represent a source of temporal heterogeneity not separately addressed; the absence of long-term follow-up likewise precludes assessment of valve durability, structural deterioration, and late clinical outcomes.

## 6. Conclusions

In this propensity score-matched cohort of 26 pairs, Su-AVR with the Perceval Plus^®^ and TAVI with self-expanding devices showed no statistically significant differences in mortality, echocardiographic outcomes, rates of postoperative atrial fibrillation and conduction disturbances, or major complication rates including low cardiac output syndrome, while TAVI offered a shorter hospital stay and Su-AVR carried a higher FFP transfusion requirement. Platelet counts declined in both groups, with a more pronounced and earlier decline in Su-AVR reflected by a significant group × time interaction (*p* = 0.013), consistent with a multifactorial mechanism involving CPB-related stress and device tissue-treatment differences in Su-AVR, glutaraldehyde fixation and procedural factors in TAVI, and a shared but unproven nitinol contribution. CRP rose in both groups, with a significantly greater and more rapid increase in the Su-AVR group (time and group × time interaction, both *p* < 0.001), an elevation that may partly relate to CPB-related processes while the more limited response in TAVI may relate to localized tissue injury and possible hypoperfusion during valve implantation, potentially amplified by patient-specific and procedural factors. Given the small sample size and residual baseline imbalances after matching, these findings warrant confirmation in larger, prospective, multicenter studies.

## Figures and Tables

**Figure 1:**
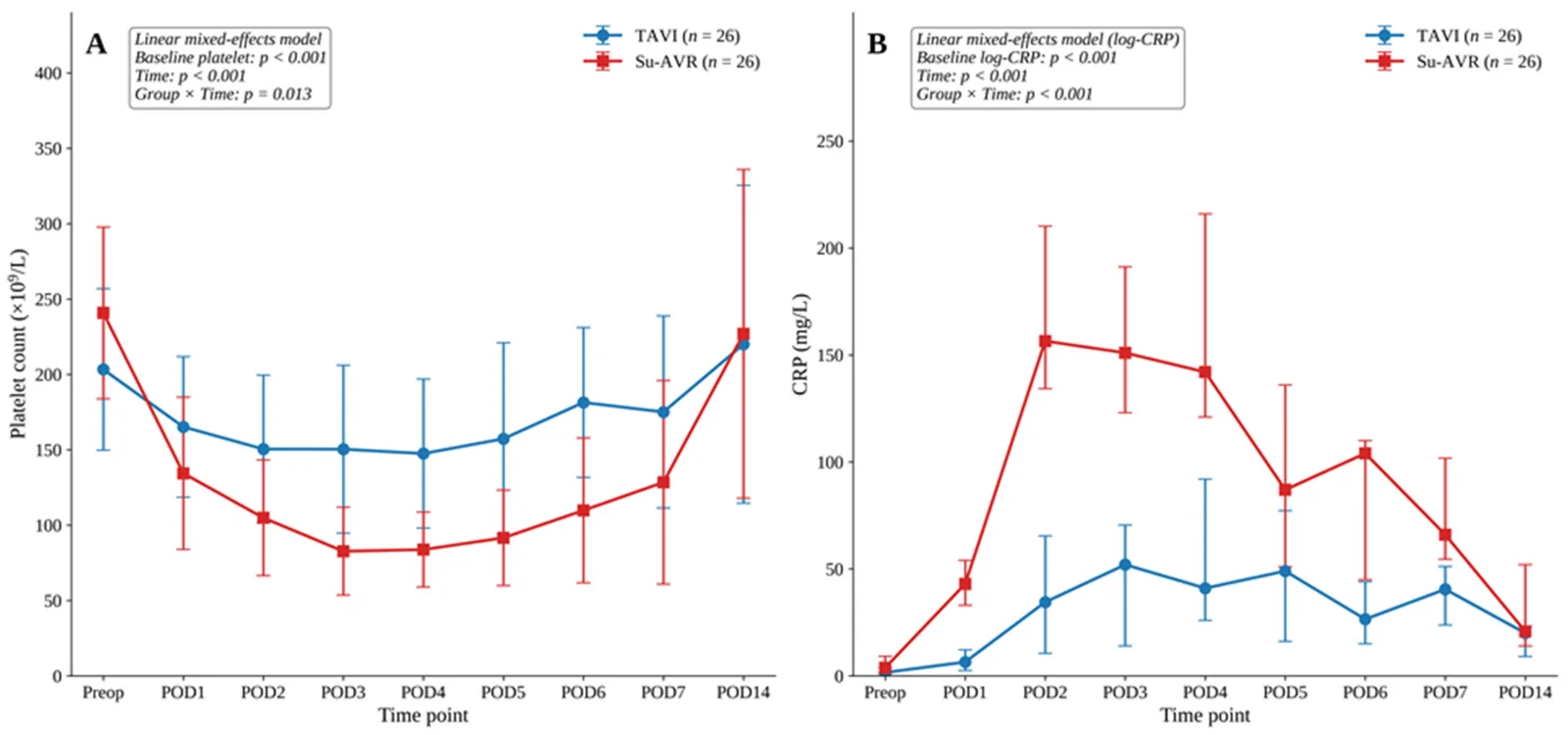
Longitudinal changes in platelet count and C-reactive protein (CRP) levels in the propensity score-matched cohort. (**A**) Platelet count (×10^9^/L), presented as mean ± SD, in the TAVI (*n* = 26) and Su-AVR (*n* = 26) groups from the preoperative period through postoperative day 14. In the linear mixed-effects model adjusted for baseline platelet count, both the time effect and the group × time interaction were statistically significant (*p* < 0.001 and *p* = 0.013, respectively). (**B**) CRP (mg/L), presented as median with interquartile range (IQR), in the same cohort and time points. CRP values were logarithmically transformed prior to analysis; in the model adjusted for baseline log-CRP, both the time effect and the group × time interaction were statistically significant (both *p* < 0.001). Error bars represent SD in (**A**) and IQR in (**B**). CRP: C-reactive protein; IQR: interquartile range; POD: postoperative day; SD: standard deviation; TAVI: transcatheter aortic valve implantation; Su-AVR: sutureless aortic valve replacement.

**Table 1:** Baseline characteristics of the full unmatched study population.

Characteristics	TAVI (*n* = 101)	Su-AVR (*n* = 53)	*p*-Value	SMD
Sex			0.611	0.09
Female	49 (48.5)	28 (52.8)		
Male	52 (51.5)	25 (47.2)		
Age (years)	77 (72–81)	70 (66–73)	<0.001	0.98
Diabetes mellitus	34 (33.7)	23 (43.4)	0.235	0.20
COPD	15 (14.9)	1 (1.9)	0.012	0.48
Chronic renal failure	16 (15.8)	7 (13.2)	0.663	0.07
Dialysis dependent	0 (0)	2 (3.8)	0.117	0.28
Prior cardiac operation	15 (14.9)	4 (7.5)	0.190	0.23
EuroSCORE II	5.6 ± 1.8	4.4 ± 1.6	<0.001	0.68
LVEF (%)	47.4 ± 8.9	52.8 ± 9.7	<0.001	0.59
Aortic valve MaxPG (mmHg)	73.4 ± 22.5	68.3 ± 23.0	0.192	0.22
Aortic valve MeanPG (mmHg)	44.6 ± 14.9	40.0 ± 14.5	0.066	0.31
Pulmonary hypertension (>45 mmHg)	17 (16.8)	1 (1.9)	0.006	0.47
Preoperative RBBB	1 (1.0)	0 (0)	1.000	0.14
Atrial fibrillation	18 (17.8)	2 (3.8)	0.014	0.42
Preoperative LBBB	9 (8.9)	1 (1.9)	0.166	0.29
Preoperative paced rhythm	1 (1.0)	0 (0)	1.000	0.14
Hypersplenism	0 (0)	0 (0)	NA	0.00
Preoperative thrombocytopenia	6 (5.9)	2 (3.8)	0.715	0.10
Liver cirrhosis	1 (1.0)	1 (1.9)	1.000	0.08
Preoperative Hb (g/dL)	11.7 ± 2.0	12.9 ± 2.0	<0.001	0.60
Preoperative leukocyte (×10^9^/L)	7.0 (6–8)	7.9 (6.6–8.7)	0.037	0.17
Preoperative Hct (%)	36.8 ± 5.9	38.7 ± 6.5	0.076	0.31
Preoperative platelet (×10^9^/L)	227.3 ± 78.3	234.5 ± 64.4	0.566	0.10
Preoperative albumin (g/L)	41.0 (38–43)	43.1 (41–45.1)	0.001	0.53
Preoperative AST (U/L)	18 (15–22)	19 (15–25)	0.550	0.08
Preoperative ALT (U/L)	14 (11–19)	15 (10–23)	0.408	0.13
Preoperative urea (mg/dL)	40.5 (31–54.5)	39.0 (31–44)	0.175	0.11
Preoperative creatinine (mg/dL)	0.9 (0.7–1.2)	0.9 (0.7–1.0)	0.152	0.12
Preoperative INR	1.0 (1.0–1.1)	1.0 (0.9–1.0)	0.008	0.36
Preoperative CRP (mg/L)	2.0 (1.0–6.8)	3.5 (1.3–12.0)	0.103	0.23

*p*-values: statistically significant (*p* < 0.05). SMD: standardized mean difference; SMD ≥ 0.2 indicates potentially meaningful imbalance. Categorical variables: *n* (%); continuous variables: mean ± SD or median (IQR). NA: not applicable. COPD: chronic obstructive pulmonary disease; LVEF: left ventricular ejection fraction; MaxPG: maximum pressure gradient; MeanPG: mean pressure gradient; RBBB: right bundle branch block; LBBB: left bundle branch block; Hb: hemoglobin; Hct: hematocrit; AST: aspartate aminotransferase; ALT: alanine aminotransferase; INR: international normalized ratio; CRP: C-reactive protein.

**Table 2:** Baseline demographic and clinical characteristics of the propensity score-matched cohort.

Characteristics	TAVI (*n* = 26)	Su-AVR (*n* = 26)	SMD
Sex			0.23
Female	15 (57.7)	12 (46.2)	
Male	11 (42.3)	14 (53.8)	
Age (years)	72 (69–78)	71.5 (69–76)	0.04
Diabetes mellitus	9 (34.6)	12 (46.2)	0.23
COPD	0 (0)	1 (3.8)	0.28
Chronic renal failure	3 (11.5)	5 (19.2)	0.21
Dialysis dependent	0 (0)	2 (7.7)	0.41
Prior cardiac operation	6 (23.1)	1 (3.8)	0.56
EuroSCORE II	4.8 ± 1.4	4.5 ± 1.4	0.21
LVEF (%)	51.1 ± 8.0	50.4 ± 8.8	0.08
Aortic valve MaxPG (mmHg)	76.3 ± 28.3	69.5 ± 24.1	0.26
Aortic valve MeanPG (mmHg)	47.8 ± 17.8	40.2 ± 15.0	0.46
Pulmonary hypertension (>45 mmHg)	5 (19.2)	1 (3.8)	0.47
Preoperative RBBB	0 (0)	0 (0)	0.00
Atrial fibrillation	3 (11.5)	0 (0)	0.51
Preoperative LBBB	2 (7.7)	1 (3.8)	0.17
Preoperative paced rhythm	0 (0)	0 (0)	0.00
Preoperative thrombocytopenia	1 (3.8)	0 (0)	0.28
Hypersplenism	0 (0)	0 (0)	0.00
Liver cirrhosis	0 (0)	0 (0)	0.00
Preoperative Hb (g/dL)	11.6 ± 2.1	12.7 ± 1.6	0.59
Preoperative leukocyte (×10^9^/L)	6.7 ± 1.0	7.7 ± 1.8	0.69
Preoperative Hct (%)	36.6 ± 6.3	38.7 ± 4.1	0.40
Preoperative platelet (×10^9^/L)	203.3 ± 53.6	240.8 ± 57.0	0.68
Preoperative albumin (g/L)	41.7 (38–44)	43.0 (41–44.8)	0.34
Preoperative AST (U/L)	18 (15–21)	17 (14–20)	0.07
Preoperative ALT (U/L)	13 (11–19)	12 (10–22)	0.09
Preoperative urea (mg/dL)	36.5 (25–41)	38.5 (32–44)	0.16
Preoperative creatinine (mg/dL)	0.8 (0.7–1.0)	0.9 (0.6–1.0)	0.12
Preoperative INR	1.0 (1.0–1.0)	1.0 (0.9–1.0)	0.18
Preoperative CRP (mg/L)	1.6 (1.0–3.8)	3.8 (1.4–9.2)	0.43

Categorical variables are presented as *n* (%), and continuous variables as mean ± standard deviation or median (interquartile range, IQR). Covariate balance was assessed using standardized mean differences (SMD), with lower SMD values indicating better balance between groups. Abbreviations are as in Table 1.

**Table 3:** Postoperative clinical outcomes in the propensity score-matched cohort.

Variables	TAVI (*n* = 26)	Su-AVR (*n* = 26)	*p*-Value
**Cardiac rhythm outcomes**			
New-onset postoperative atrial fibrillation	0 (0)	5 (19.2)	0.063 *
Postoperative LBBB	2 (7.7)	1 (3.8)	1.000 *
Postoperative RBBB	0 (0)	0 (0)	NA
Complete atrioventricular block	0 (0)	1 (3.8)	1.000 *
**Mortality**			
30-day mortality	1 (3.8)	1 (3.8)	1.000 *
In-hospital mortality	1 (3.8)	2 (7.7)	1.000 *
**Hospital course**			
ICU stay (days) **	2 (2–2)	2 (2–4)	0.041
Hospital stay (days)	6 (5–8)	10 (8–12)	0.016
**Transfusion requirements**			
RBC transfusion (units)	0 (0–1)	0 (0–1)	0.134
FFP transfusion (units)	0 (0–0)	0 (0–1)	0.016
Platelet transfusion (units)	0 (0–0)	0 (0–0)	0.317
**Postoperative complications**			
Low cardiac output syndrome	0 (0)	1 (3.8)	1.000 *
Re-exploration for bleeding	0 (0)	0 (0)	NA
Respiratory insufficiency	0 (0)	0 (0)	NA
Gastrointestinal bleeding	1 (3.8)	0 (0)	1.000 *
Renal replacement therapy	0 (0)	1 (3.8)	1.000 *
Hemorrhagic stroke	0 (0)	0 (0)	NA
Ischemic stroke	0 (0)	0 (0)	NA
**Echocardiographic outcomes**			
Maximum aortic valve gradient (mmHg)	11 (9–18)	13.5 (11–16.5)	0.130
Mean aortic valve gradient (mmHg)	5 (5–8)	7 (5–9)	0.107
Paravalvular leak	3 (11.5)	1 (3.8)	0.625 *

Categorical variables compared using exact McNemar test (* denotes exact McNemar test); continuous variables compared using Wilcoxon signed-rank test. ** For ICU stay, the corresponding mean ± SD values were 2.19 ± 1.06 days in the TAVI group and 4.62 ± 8.41 days in the Su-AVR group. Data: *n* (%) or median (IQR). NA: not applicable. ICU: intensive care unit; RBC: red blood cells; FFP: fresh frozen plasma; LBBB: left bundle branch block; RBBB: right bundle branch block.

**Table 4:** Longitudinal changes in platelet count and C-reactive protein levels in the propensity score-matched cohort.

Variables	TAVI (*n* = 26)	Su-AVR (*n* = 26)	*p*-Value
**Platelet count (×10^9^/L), mean ± SD**			
Preoperative	203.3 ± 53.6	240.8 ± 57.0	
Postoperative day 1	165.2 ± 46.6	134.4 ± 50.5	
Postoperative day 2	150.5 ± 49.0	104.9 ± 38.4	
Postoperative day 3	150.4 ± 55.7	82.7 ± 29.2	
Postoperative day 4	147.5 ± 49.4	83.8 ± 24.9	
Postoperative day 5	157.3 ± 63.7	91.6 ± 31.7	
Postoperative day 6	181.4 ± 49.7	109.8 ± 48.0	
Postoperative day 7	175.1 ± 63.7	128.4 ± 67.5	
Postoperative day 14	220.0 ± 105.4	226.9 ± 109.0	
Baseline platelet			<0.001
Time			<0.001
Group × Time			0.013
**CRP (mg/L), median (IQR)**			
Preoperative	1.6 (1.0–3.8)	3.8 (1.4–9.2)	
Postoperative day 1	6.5 (2.6–12.2)	43.0 (33.0–54.0)	
Postoperative day 2	34.5 (10.5–65.5)	156.5 (134.2–210.2)	
Postoperative day 3	52.0 (14.0–70.5)	151.0 (123.0–191.2)	
Postoperative day 4	41.0 (26.0–92.0)	142.0 (121.0–216.0)	
Postoperative day 5	49.0 (16.2–77.2)	87.0 (51.0–136.0)	
Postoperative day 6	26.5 (15.0–44.0)	104.0 (45.0–110.0)	
Postoperative day 7	40.5 (23.8–51.2)	66.0 (54.5–101.8)	
Postoperative day 14	20.0 (9.1–23.2)	20.8 (14.0–52.0)	
Baseline log-CRP			<0.001
Time			<0.001
Group × Time			<0.001

Platelet: mean ± SD; CRP: median (IQR). Postoperative platelet levels were analyzed using a linear mixed-effects model adjusted for baseline platelet count. CRP values were logarithmically transformed because of their right-skewed distribution, and the longitudinal CRP model was adjusted for baseline log-transformed CRP. Time was modeled as a categorical fixed effect; group, time, and group × time interaction were included as fixed effects, with subject included as a random intercept using a variance component covariance structure. CRP: C-reactive protein.

**Table 5:** Procedural and postoperative treatment characteristics of the matched cohort.

Variables	TAVI (*n* = 26)	Su-AVR (*n* = 26)
**Concomitant procedures**		
CABG	—	1 (3.8)
Mitral valve repair	—	0 (0)
Mitral valve replacement	—	0 (0)
Tricuspid valve annuloplasty	—	0 (0)
Ascending aorta replacement	—	0 (0)
LVAD	—	0 (0)
**Surgical approach**		
Median sternotomy	—	20 (76.9)
Minimally invasive	—	6 (23.1)
**Su-AVR procedural characteristics**		
**Valve size**		
Small	—	6 (23.1)
Medium	—	13 (50.0)
Large	—	6 (23.1)
Extra Large	—	1 (3.8)
CPB time (min)	—	70.5 (50–81)
Cross-clamp time (min)	—	48.5 (34–58)
**TAVI procedural characteristics**		
** Valve type**		
Medtronic CoreValve	17 (65.4)	—
Evolut R	8 (30.8)	—
Evolut Pro	1 (3.8)	—
Valve size (mm), median (IQR)	29 (29–29)	—
**Postoperative anticoagulation**		
None	23 (88.5)	11 (42.3)
Apixaban	3 (11.5)	2 (7.7)
Bemiparin	0 (0)	1 (3.8)
Rivaroxaban	0 (0)	5 (19.2)
Warfarin	0 (0)	6 (23.1)
Exitus	0 (0)	1 (3.8)
Postoperative antiplatelet therapy	24 (92.3)	20 (80)

Data: *n* (%) or median (IQR). Postoperative antiaggregant percentages were calculated among surviving patients with available postoperative treatment data (TAVI: *n* = 26; Su-AVR: *n* = 25). The “Exitus” category refers to the Su-AVR patient who died before a postoperative anticoagulation regimen could be established (see Discussion for mortality details). CABG: coronary artery bypass grafting; LVAD: left ventricular assist device; CPB: cardiopulmonary bypass. —: not applicable (procedure or device not relevant to the respective treatment modality).

## Data Availability

The data generated in this study are available from the corresponding author upon reasonable request.

## Supplementary Materials

The following supporting information can be downloaded at: https://www.mdpi.com/article/10.3390/life16101606/s1, Table S1: Expanded propensity-score matching sensitivity analysis; Table S2: Baseline characteristics of matched and excluded TAVI patients; Table S3: Number of available platelet and CRP measurements at each time point in the propensity score–matched cohort; Table S4: Baseline-adjusted between-group differences in postoperative platelet and CRP levels in the propensity score–matched cohort; Table S5: Covariate balance and longitudinal sensitivity analyses after inverse probability of treatment weighting in the full cohort.

## Abbreviations

**AS**	Aortic Stenosis
**CI**	Confidence Interval
**SAVR**	Surgical Aortic Valve Replacement
**CPB**	Cardiopulmonary Bypass
**Su-AVR**	Sutureless Aortic Valve Replacement
**TAVI**	Transcatheter Aortic Valve Implantation
**FREE**	Phospholipid reduction + aldehyde neutralization + aldehydE. In Corcym’s own publications, this process has not been clearly defined and is generally referred to directly as “FREE anticalcification treatment” in the literature.
**XL**	Extra Large
**CRP**	C-Reactive Protein
**S.r.l.**	Società a responsabilità limitata
**USA**	United States of America
**UK**	United Kingdom
**MN**	Minnesota
**AVR**	Aortic Valve Replacement
**CABG**	Coronary Artery Bypass Grafting
**LVAD**	Left Ventricular Assist Device
**COPD**	Chronic Obstructive Pulmonary Disease
**AF**	Atrial Fibrillation
**LBBB**	Left Bundle Branch Block
**RBBB**	Right Bundle Branch Block
**PH**	Pulmonary Hypertension
**LVEF**	Left Ventricular Ejection Fraction
**MaxPG**	Maximum Pressure Gradient
**MeanPG**	Mean Pressure Gradient
**PVL**	Paravalvular Leak
**EuroSCORE II**	European System for Cardiac Operative Risk Evaluation II
**Hb**	Hemoglobin
**Hct**	Hematocrit
**AST**	Aspartate Aminotransferase
**SGOT**	Serum Glutamic-Oxaloacetic Transaminase (Aspartate Aminotransferase)
**ALT**	Alanine Aminotransferase
**SGPT**	Serum Glutamic-Pyruvic Transaminase (Alanine Aminotransferase)
**INR**	International Normalized Ratio
**ICU**	Intensive Care Unit
**RBC**	Red Blood Cells
**FFP**	Fresh Frozen Plasma
**RRT**	Renal Replacement Therapy
**PSM**	Propensity Score Matching
**SMD**	Standardized Mean Difference
**SD**	Standard Deviation
**IQR**	Interquartile Range
**LME**	Linear Mixed-Effects Model
**SPSS**	Statistical Package for the Social Sciences
**IBM**	International Business Machines Corporation
**NA**	Not Applicable
**NY**	New York
**HCA**	Homocysteic Acid
**SIRS**	Systemic Inflammatory Response Syndrome
**TF-TAVI**	Transfemoral Transcatheter Aortic Valve Implantation
**IL-6**	Interleukin-6
**IL-8**	Interleukin-8
