# Supplementary material for: Sutureless Aortic Valve Replacement with the Perceval Plus® Versus TAVI: A Propensity-Matched Analysis of Postoperative Outcomes, Hematological Dynamics, and Inflammatory Response
Source: Life (Basel). 2026 Sep 25;16(10):1606. doi: 10.3390/life16101606 (PMC13706003; doi:10.3390/life16101606)
Supplement: Supplementary file 1 [file life-16-01606-s001.zip › life-4544231-supplementary.pdf]

Supplementary Table S1. Expanded propensity-score matching sensitivity analysis

Covariate / outcome	Original PSM (26 pairs)	Expanded PSM sensitivity (35 pairs)
Age	0.040	0.032
Sex	0.230	0.118
LVEF	0.080	0.061
EuroSCORE	0.210	0.019
Chronic renal failure	0.210	0.085
COPD	0.280	0.000
Preoperative platelet	0.680	0.059
Preoperative leukocyte	0.690	0.074
Preoperative CRP	0.430	0.081
Longitudinal sensitivity analysis		
Platelet: group x time p-value	0.013	0.009
CRP: group x time p-value	<0.001	<0.001

The expanded propensity-score model included age, sex, LVEF, EuroSCORE, chronic renal failure, COPD, baseline platelet count, baseline leukocyte count, and baseline CRP. One-to-one nearest-neighbor matching without replacement was performed using a 0.2-SD caliper of the logit of the propensity score, yielding 35 matched pairs. Longitudinal models were adjusted for the corresponding baseline value and treated time as a categorical variable. CRP was log-transformed.

Supplementary Table S2. Baseline characteristics of matched and excluded TAVI patients

Variable	Matched TAVI (n=26)	Excluded/unmatched TAVI (n=75)	SMD
Age (years)	72.0 (69.0–78.0)	78.0 (73.5–83.0)	0.782
Sex, male	11 (42.3)	41 (54.7)	0.249
LVEF (%)	51.1±8.0	46.1±8.8	0.586
EuroSCORE	4.8±1.4	5.9±1.9	0.668
Chronic renal failure	3 (11.5)	13 (17.3)	0.165
COPD	0 (0.0)	15 (20.0)	0.707
Preoperative platelet	203.3±53.6	235.7±84.1	0.460
Preoperative leukocyte	6.7±1.0	7.8±3.2	0.455
Preoperative CRP	1.6 (1.0–3.8)	2.9 (1.0–6.9)	0.264
Pulmonary hypertension (>45 mmHg)	5 (19.2)	12 (16.0)	0.085
Atrial fibrillation	3 (11.5)	15 (20.0)	0.234
Preoperative Hb	11.6±2.1	11.7±1.9	0.039

Data are presented as n (%), mean ± standard deviation, or median (interquartile range), consistent with the main tables. SMD: standardized mean difference. Larger absolute SMD values indicate greater differences between matched and excluded TAVI patients.

Supplementary Table S3. Number of available platelet and CRP measurements at each time point in the propensity score–matched cohort

Time	Platelet TAVI, n	Platelet Su-AVR, n	CRP TAVI, n	CRP Su-AVR, n

Preoperative	26	26	26	26
POD1	26	25	26	25
POD2	25	24	24	24
POD3	19	20	15	12
POD4	16	20	13	13
POD5	12	17	10	11
POD6	10	14	12	9
POD7	9	16	8	12
POD14	9	18	8	17

Values indicate the number of patients with available measurements at each time point. The propensity score–matched cohort included 26 patients in each treatment group. POD: postoperative day; CRP: C-reactive protein.

Supplementary Table S4. Baseline-adjusted between-group differences in postoperative platelet and CRP levels in the propensity score–matched cohort

Outcome / Time	Adjusted effect estimate	95% CI	p-value
**Platelet, adjusted mean difference (TAVI − Su-AVR)**			
POD1	**+49.9**	**23.3–76.5**	**<0.001**
POD2	**+65.3**	**38.2–92.4**	**<0.001**
POD3	**+80.9**	**51.5–110.3**	**<0.001**
POD4	**+78.6**	**47.8–109.4**	**<0.001**
POD5	**+71.4**	**37.8–105.1**	**<0.001**
POD6	**+68.3**	**31.6–104.9**	**<0.001**
POD7	**+55.9**	**18.9–92.9**	**0.003**
POD14	+6.2	−30.2–42.5	0.740
**CRP, adjusted ratio (TAVI/Su-AVR)**			
POD1	**0.15**	**0.09–0.25**	**<0.001**
POD2	**0.19**	**0.12–0.31**	**<0.001**
POD3	**0.32**	**0.17–0.61**	**<0.001**
POD4	**0.51**	**0.27–0.97**	**0.039**
POD5	0.68	0.34–1.36	0.275
POD6	**0.42**	**0.21–0.84**	**0.014**
POD7	0.67	0.33–1.38	0.276
POD14	0.74	0.37–1.45	0.380

Platelet estimates are adjusted mean differences (TAVI − Su-AVR) derived from a linear mixed-effects model adjusted for baseline platelet count. CRP was logarithmically transformed because of its right-skewed distribution; CRP estimates represent exponentiated adjusted between-group contrasts (TAVI/Su-AVR) derived from the model adjusted for baseline log-transformed CRP. Time was modeled as a categorical fixed effect, and subject was included as a random intercept. Positive platelet differences indicate higher adjusted platelet levels in the TAVI group. CRP ratios <1 indicate lower adjusted CRP levels in the TAVI group relative to the Su-AVR group. CI: confidence interval; CRP: C-reactive protein; POD: postoperative day.

Supplementary Table S5. Covariate balance and longitudinal sensitivity analyses after inverse probability of treatment weighting in the full cohort

Variable / outcome	Unweighted SMD	IPTW-weighted SMD	p-value
**Covariate balance**			
Age	0.982	0.143	-
Sex	0.086	0.031	-
LVEF	0.585	0.115	-
EuroSCORE	0.678	0.039	-
Chronic renal failure	0.075	0.094	-
COPD	0.482	0.352	-
**Longitudinal sensitivity analysis**			
**Platelet**			
Time	-	-	**<0.001**
Group x Time	-	-	**0.024**
**CRP**			
Time	-	-	**<0.001**
Group x Time	-	-	**<0.001**

IPTW sensitivity analyses were performed in the full cohort (N=154; TAVI, n=101; Su-AVR, n=53). Propensity scores were estimated using age, sex, LVEF, EuroSCORE, chronic renal failure, and COPD, consistent with the propensity score matching model. Stabilized inverse probability of treatment weights were applied and truncated at the 1st and 99th percentiles to reduce the influence of extreme weights. Covariate balance was assessed using standardized mean differences (SMD), with lower absolute SMD values indicating improved balance. Longitudinal outcomes were analyzed using weighted generalized estimating equations with robust standard errors, assuming an independence working correlation structure, with time modeled as a categorical variable. The platelet model was adjusted for baseline platelet count. CRP was logarithmically transformed because of its right-skewed distribution, and the CRP model was adjusted for baseline log-transformed CRP. IPTW: inverse probability of treatment weighting; SMD: standardized mean difference; LVEF: left ventricular ejection fraction; COPD: chronic obstructive pulmonary disease; CRP: C-reactive protein.
